# Practice, Knowledge, and Attitude of Health Care Providers regarding Cancer Pain Management: A National Survey

**DOI:** 10.1155/2021/1247202

**Published:** 2021-08-23

**Authors:** Jinmei Liu, Ming Zhang, Juan Luo, Jiyi Xie, Xu Chen, Hanxiang Wang, Shijun Li, Shengli Yang, Chunfen Peng, Liangle Yang, Bin Deng, Yu Zhang, Cong Wang, Jianli Hu, Chen Shi

**Affiliations:** ^1^Department of Pharmacy, Union Hospital, Tongji Medical College, Huazhong University of Science and Technology, Wuhan 430022, China; ^2^Hubei Province Clinical Research Center for Precision Medicine for Critical Illness, Wuhan 430022, China; ^3^President's Office of Union Hospital, Tongji Medical College, Huazhong University of Science and Technology (HUST), Wuhan, China; ^4^Cancer Center, Union Hospital, Tongji Medical College, Huazhong University of Science and Technology, Wuhan, China; ^5^Department of Occupational and Environmental Health, Ministry of Education and Ministry of Environmental Protection, and Key Laboratory of Environmental Health, School of Public Health, Tongji Medical College, Huazhong University of Science and Technology, Wuhan, China

## Abstract

**Background:**

A lack of knowledge and inadequate practices of health care providers (HCPs) are the main obstacles to effective cancer pain management (CPM). The main objective of the study was to evaluate the CPM knowledge, CPM practice, and attitudes towards pharmacists' participation and advanced methods in CPM of physicians, nurses, and pharmacists in China.

**Methods:**

An open online survey was adopted using social media software (WeChat) as the platform to conduct a nationwide survey of HCPs involved in CPM in public medical institutions at all levels in China from March to June 2019.

**Results:**

A total of 1279 physicians, 2267 nurses, and 1466 pharmacists participated in the survey. Among the three types of professionals, nurses had the highest level of practical ability (61.63 ± 28.99) and best attitudes towards pharmacists' participation and advanced methods in CPM (72.05 ± 33.71) and physicians had the best mastery of CPM-related knowledge (69.60 ± 28.45), while pharmacists performed the worst in these three aspects (50.04 ± 26.69, 61.49 ± 28.95, and 62.07 ± 36.46, respectively). Only 19.69% of the hospitals had a pharmacist to tumor patient ratio ≥1 : 50. Hierarchical analysis showed that passing a good pain management (GPM) ward program and participating in advanced training had positive impacts on the scores of all three parts in the three professions (*p*trend <0.05).

**Conclusions:**

HCPs' levels of practice, knowledge, and attitudes towards pharmacists and advanced methods of CPM were average in China; however, pharmacists had the worst performance, which demonstrates a need for further improvement. Furthermore, GPM ward programs and advanced trainings are helpful for improving CPM levels.

## 1. Introduction

Pain is highly prevalent among patients with cancer, especially those with advanced or metastatic cancer [1]. Adequate pain assessment and management is essential to improve their quality of life, emotional well-being, relationships, and health outcomes [2]. In 2018, 20% of new cancer cases in the world were reported in China [3], making pain management an important topic in China.

Cancer pain can be effectively reduced through appropriate drug treatment [4]; however, 39.1%∼60% of patients with cancer have not received adequate analgesic treatment or are not satisfied with the treatment [5–8]. Undertreatment has been ascribed to some confounding factors that exist in all aspects of health care providers (HCPs), patients, and related systems [9]. Surveys of HCPs may help elucidate the barriers to the adoption of best practices in cancer pain management. All over the globe, numerous studies have evaluated the knowledge and attitude of HCPs towards cancer pain management (CPM); however, many of these studies focused only on a certain type of HCP [10–13]. In China, the literature exploring HCPs' attitudes and knowledge regarding pain management is very limited, and it mainly focuses on physicians or nurses [14, 15]. Most reports have indicated that HCPs (particularly nurses) generally lack knowledge of CPM [10, 11, 16]. Only a few studies decades ago have evaluated knowledge and attitudes toward CPM of pharmacists, and the findings demonstrated that pharmacists also had misconceptions about CPM [17–19]. It is noteworthy that there is no study concurrently comparing Chinese physicians, pharmacists, and nurses in China. Clinical pharmacists play active roles in Cancer Pain Multidisciplinary Management Teams and have gained increasing attention [20–22]. However, these reports are all based on pharmacists' self-evaluations, and their service value needs to be recognized by other clinical HCPs.

Based on all the above information, this study involved the design of a set of questionnaires to simultaneously evaluate the knowledge and practice regarding CPM of physicians, pharmacists, and nurses at different levels of public hospitals and departments in China. Unlike the existing studies with pharmacists [17–19], this study also investigated the current situation of pharmacists' participation in CPM in China and the demand of medical staff for the services of clinical pharmacists. The use of mobile management systems in CPM has gradually emerged and achieved good results [23]. This study investigated the demand of HCPs for advanced management methods (e.g., mobile CPM systems), and the results can provide a reference for improvements to the design and development of related products in the future.

As China has started to promote the WHO “3-step analgesic ladder” Program and launch the good pain management (GPM) ward program, some progress has been achieved in CPM [24]. Nevertheless, there is a long way to go before achieving the goal of the pain-free management of patients with cancer. As the first national large-sample cross-sectional survey of physicians, nurses, and pharmacists, this investigation studied the knowledge, practice, and attitude of HCPs and the existing problems regarding CPM in China and thereby provides the basis for the future targeted improvement of CPM.

## 2. Materials and Methods

### 2.1. Participants

This cross-sectional online survey was conducted in public medical institutions at all levels in China from March to June 2019. All HCPs from the Departments of Medical Oncology, Surgical Oncology, Pain, Anesthesiology, etc. involved in CPM were eligible for participation. The survey utilized the Blue Ribbon Cancer Pain Patients Care Cooperation Group as the promoter, recruiting volunteers through the WeChat online social media platform or via face-to-face communication in the palliative care training sessions. Furthermore, the survey invited 1 national well-known clinical leader and 14 local well-known experts in this field to circulate the questionnaire via their networks, to effectively distribute it throughout the country. All participation in the survey was voluntary and anonymous. Approximately 10–15 minutes was required to complete the questionnaire on the online platform Questionnaire Star (http://www.wjx.cn/), and only after all questions were completed online as required could the questionnaire be submitted. Therefore, any responding questionnaire successfully received by the system was valid. As an open online survey, the participants voluntarily participated, potentially leading to nonrepresentativeness of the respondents. In this study, an overrepresentation of motivated respondents with experience of, and interest in, CPM were considered to be supportive of, rather than disabling to, our aims.

### 2.2. Survey Instrument

The questionnaire was created by researchers according to the purpose and requirements of the survey and revised by a panel of experts comprising medical oncologist (S. L. Y.), pharmacologists (Y. Z. and C. S.), nurse (C. F. P.), palliative care expert (J. L. H.), and epidemiologist (L. L. Y.). The piloted survey was carried out in one hospital by a small number of HCPs (*n* = 9; 2 physicians, 2 nurses, and 5 pharmacists) to assess wording, structure, layout, and readability and to provide an estimate for completion time. The pilot study resulted in very minor modifications to the word expression and data from the pilot participants were not included in the final analysis.

The questionnaire design was divided into the following four parts with a total of 30 questions: (1) basic information; (2) CPM practical ability; (3) knowledge about CPM; and (4) attitudes towards pharmacists' participation and advanced methods in CPM. The first section consisted of 9 questions on background characteristics of the sample including occupation, professional title, religion, hospital grade, and departments. The second section contained 6 questions for assessing the practice of HCPs in CPM from the follow-up frequency of discharged cancer pain patients, the use of pain assessment tools, the timing of pain assessment, the notification of adverse reactions, and the choice of analgesics. In the third section, knowledge of CPM was evaluated through 6 items (3 multiple-choice questions and 3 Likert-scale questions), tapping into the mastery of the guidelines, opioid dose calculation, the specific properties of analgesics, and the principles of CPM. The final section consisted of 9 questions (2 single-choice questions, 3 multiple-choice questions, and 4 Likert-scale questions) to assess HCPs' attitudes towards pharmacists and advanced CPM methods. Participants were asked to indicate whether there were pharmacists participating in CPM in their hospital, in what forms, and their needs for pharmacists and advanced CPM methods in their work.

Several questions in the questionnaire were answered by specific occupational groups. Question 6 (“What proportion of patients under your care had cancer-related pain?”) was for doctors and nurses only, while questions 14 (“What are the commonly used analgesics in your analgesic treatment?”) and 15 (“What are the reasons why you prefer to use these drugs?”) were for doctors only. Cronbach's *α* for the questionnaire was 0.89, 0.84, and 0.83 for physicians, nurses, and pharmacists, respectively, indicating acceptable internal consistency.

### 2.3. Data Processing

In addition to the 9 basic information items, 7 of the remaining 21 items were used for descriptive analysis (Supplementary Figures 1–4), and the remaining 14 questions (Table 1) were used to calculate scores to evaluate participants' performance in all the three aspects. These 14 items were divided into 2 types: Likert-scale questions and multiple-choice questions. Data analysis was divided by question type: ① Likert-scale questions, which were scored according to the Likert-scale (degree of agreement or correctness); for example, if the answer contains 4 options, the lowest score is 0 and the highest score is 3; and ② multiple-choice questions, which were scored according to the number of choices; for example, if the choice was correct, the score was +1; if not, the score was −1. This would reduce the impact of participants guessing answers. If there were 5 choices in total, then the score range was −5 to +5, and if there were 6 choices, the score range was −6 to +6, and so on.

To facilitate analysis, all scores were normalized to a 0–100 numeric rating scale.

1. The equation used to calculate the normalized score for Likert-scale questions is normalized score = 100/ (no. of choices−1) × prenormalized score
2. The equation used to calculate the normalized score for multiple-choice questions [12] is normalized score = 50/(no. of choices) × prenormalized score +50

The overall score of the questionnaire was the average normalized score for the all the 14 items, and the aspect (practice, knowledge, and attitude) score was the average score of all of the items in each aspect. Both the total score and aspect score ranged from 0 to 100 points. The evaluation criteria for the score were as follows: <50 points was considered “poor,” 50–75 points was considered “normal,” and ≥75 points was considered “good” [25].

### 2.4. Statistical Analysis

Differences in the scores of physicians, nurses, and pharmacists were tested by using the Kruskal–Wallis nonparametric test. Binary logistic regression analysis was used to evaluate the associations between different factors and HCP scores >65 points. In this model, “score” was classified as a dummy variable (0, 1). Sixty-five points was used as a cutoff value for stratification, as the median overall score of HCPs was 63.47 points. Stratified analyses were performed to analyze the influence of six major characteristics of the study population (professional title, hospital grade, proportion of patients with cancer-related pain (for doctors and nurses only), passing of the GPM ward program, number of CPM trainings received per year, and types of CPM trainings) on the scores of participants in different occupations. All statistical analyses were performed using IBM SPSS Statistics 23.0. Results with *P* < 0.05 were considered statistically significant.

## 3. Results

### 3.1. Basic Information

A total of 5012 participants were investigated in this study, with 1279 doctors, 2267 nurses, and 1466 pharmacists. The proportion of HCPs from grades 3 and 2 and lower hospitals was 75.12%, 20.39%, and 4.49%, respectively. In China, grades 3, 2, and 1 hospitals are responsible for approximately 80%, 15%, and 5% of the diagnosis and treatment of patients with cancer, respectively [26, 27]. This means that the samples collected in this survey were quite representative. Nurses mainly had junior professional titles (53.07%), pharmacists mostly had intermediate professional titles (55.39%), and doctors had a slightly higher proportion of senior titles (14.13%) than the other two professions. Among the respondents, 53.29% and 44.95% came from grade 3 hospitals and oncology departments, respectively, 54.63% of the departments or hospitals in which they served had passed the GPM ward review, 42.98% of doctors and nurses indicated that more than 40% of the patients in their charge were cancer pain patients, 88.39% had received CPM training at least once a year, and 33.30% had received at least 3 different forms of CPM training (Table 2).

### 3.2. CPM Practical Ability

With regard to the different occupations, nurses scored the highest on practical ability (61.63 ± 28.99), followed by physicians (57.00 ± 28.74) and pharmacists (50.04 ± 26.69) (*P* < 0.01) (Table 1). It can be seen that, according to the evaluation criteria described in Section 2.3, all of them had reached the “normal” level. HCPs from northern China (OR = 0.47, 95% CI 0.36–0.61), those from departments other than medical oncology, and those who had caseloads composed of 21–40% cancer pain patients (OR = 0.57, 95% CI 0.38–0.85) were less likely to have scores >65 points. In contrast, nurses (OR = 1.77, 95% CI 1.45–2.15), HCPs who came from hospitals that had successfully passed the GPM ward review (OR = 2.16, 95% CI 1.83–2.55), HCPs who had received CPM training every year, and those who had received at least 3 different forms of CPM training were more likely to score >65 points (Table 3).

The analgesic drugs most commonly used by physicians in this survey were oxycodone hydrochloride sustained-release tablets (53.24%), morphine sustained-release tablets (45.82%), morphine immediate-release tablets (40.50%), morphine injections (36.36%), and fentanyl transdermal patches (18.45%). Additionally, the usage rate of pethidine hydrochloride (dolantin) ranked sixth, at 9.46%. The main factors that determined the analgesic drugs commonly prescribed by physicians included the following: better analgesic effect (60.99%), ease of use and acceptance by the patients (53.79%), lower incidence of adverse reactions (45.43%), higher safety (45.19%), and guideline recommendations (39.72%) (Supplementary Figure 1).

### 3.3. CPM-Related Knowledge

In terms of cognition of CPM, although the score of doctors was 69.60 ± 28.45, which was significantly higher than that of nurses 64.28 ± 28.01 and pharmacists 61.49 ± 28.95, they were still at the “normal” level (Table 1). All the factors in Table 2 affected the level of mastery of CPM-related knowledge of HCPs (Table 3). Nurses (OR = 0.49, 95% CI 0.41–0.57), pharmacists (OR = 0.58, 95% CI 0.40–0.83), HCPs from hospitals other than grade 3 first-level, and HCPs from departments other than medical oncology were less likely to score >65 points. Conversely, HCPs with more senior professional titles, those who had received CPM training every year, and those who had received at least 3 different forms of CPM training typically had scores >65 points. Moreover, it is interesting to note that the scores regarding adverse drug reactions in these three types of professionals were all lower than 50 points, which was a poor level (Table 1).

### 3.4. Attitudes towards Pharmacists' Participation and Advanced Methods in CPM

A total of 54.65% of the participants indicated that there were pharmacists participating in CPM, and only 19.69% of them indicated that the ratio of pharmacists involved in CPM to cancer patients reached 1:1–50 (Supplementary Figure 2). Pharmacists' participation in CPM mainly involves rational drug usage guidance (70.43%), ward rounds (56.81%), drug usage monitoring (53.01%), consultation for difficult cases (46.40%), drug usage training (38.85%), dissemination of information regarding drug usage to the public (38.59%), and case review (37.57%) (Supplementary Figure 3).

The overall scores for physicians, nurses, and pharmacists' attitudes towards pharmacists' participation and advanced methods in CPM were 70.35 ± 34.43, 72.05 ± 33.71, and 62.07 ± 36.46, respectively (*P* < 0.01) (Table 1), which were all at the “normal” level, indicating that physicians and nurses perceived a greater need for pharmacists and advanced CPM methods than pharmacists. HCPs who came from grade 3 first-level hospitals (OR = 1.52, 95% CI 1.10–2.10), who came from hospitals that successfully passed the GPM ward review (OR = 1.18, 95% CI 1.59–2.07), who had received training 1–3 times a year (OR = 1.31, 95% CI 1.06–1.61), and who had received at least 3 different forms of CPM training were more likely to score >65 points (Table 3).

Regarding the functional requirements of the management system, HCPs had the highest requirements for six functions: automatic screening; identifying pain patients; online pain assessments; intelligent delivery of pain orders; online conversion of different opioid dosages; online communication between physicians, pharmacists, and nurses; and regular delivery of popular science articles (Supplementary Figure 4).

### 3.5. Hierarchical Analysis of the Factors Influencing the Scores of Different Professions

**Figure 1:**
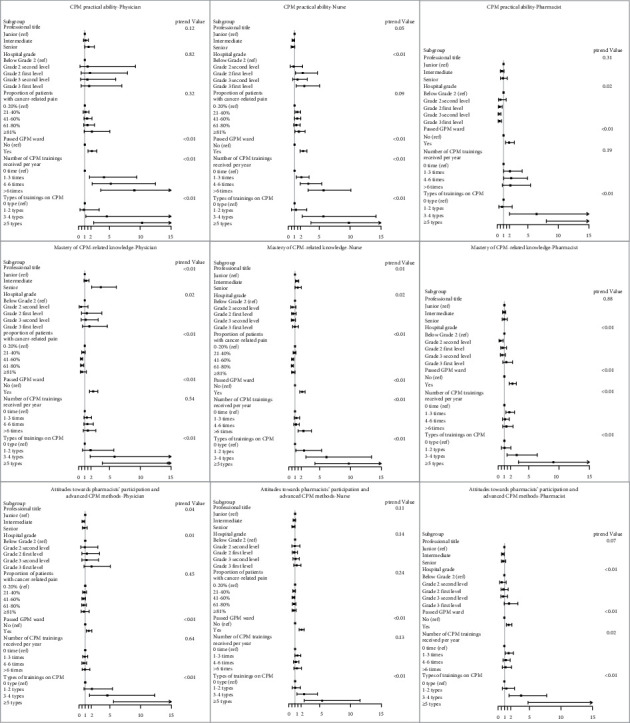
Adjusted odds ratios for HCPs' practice, knowledge, and attitude scores greater than 65 points stratified by occupation. Each of the six variables of professional title, hospital grade, proportion of patients with cancer‐related pain (for physicians and nurses only), whether the hospital had passed the GPM ward, number of CPM trainings received per year, and types of training on CPM were adjusted for the other basic information covariates except itself and occupation.

Stratified analyses were subsequently conducted to analyze the influence of six major characteristics of the study population (Figure 1) on the scores of participants in different occupations. The findings demonstrate that working in a hospital that has passed the GPM ward review and receiving various forms of training had significant positive impacts on the scores on all four parts of the survey in the three professions (*p*trend <0.05). However, the remaining three factors had heterogeneous effects on the scores on the three parts.

## 4. Discussion

In this study, four aspects of the practical ability of HCPs were investigated: the timing of pain assessment, assessment tools, analgesic adverse drug reaction notification, and follow-up frequency for discharged cancer pain patients. The average overall scores for physicians, nurses, and pharmacists were 57.00 ± 28.74 vs. 61.63 ± 28.99 vs. 50.04 ± 26.69 (*P* < 0.01), respectively (Table 1). Nurses scored the highest and performed the best on each item (Table 1), which was consistent with the literature [16, 17, 19]. This may be due to the division of labor of physicians, pharmacists, and nurses. In clinical work, nurses are the group most closely and continuously in contact with patients. Monitoring patients' physical signs (including pain) is one of the main responsibilities of nurses. These findings also reflected the shortcomings of insufficient pain assessment, the nonstandard use of assessment tools, and irregular discharge follow-up in CPM in China, suggesting that HCPs should strengthen pain assessments to make them more comprehensive and dynamic and should strive to extend CPM to outpatient follow-up. In terms of the mastery of CPM-related knowledge, although doctors scored higher than nurses and pharmacists (69.60 ± 28.45 vs. 64.28 ± 28.01, 61.49 ± 28.95 (*P* < 0.01)) (Table 1), all of them had only general knowledge of CPM, which is consistent with previous study results [12, 15, 16, 19]. Only a few studies found an adequate level of knowledge of CPM in HCPs [28, 29]. A large number of other studies have shown that HCPs, especially nurses, generally had insufficient CPM knowledge, which was an important obstacle to pain management, especially in Europe, Africa, and Asia [12, 25, 30, 31]. HCPs' knowledge of cancer pain has also been found to have regional differences. For example, the average score for nurses in the United States was 86.4%, followed by Turkey, Italy, and Jordan, which were 53.8%, 54.1%, and 51.5%, respectively [25, 27, 32, 33]. These findings suggest that the knowledge of CPM in Chinese physicians, pharmacists, and nurses is at an intermediate level.

Interestingly, pharmacists' scores for specific problems or pharmacological problems (question 21) were significantly higher than those of physicians and nurses (80.43 ± 15.93 vs. 77.85 ± 18.26, 75.44 ± 18.16, *P* < 0.01) (Table 1), which was consistent with the results of the study by Xue et al. [19]. CPM should be addressed with a multidisciplinary collaborative management team composed of physicians, pharmacists, and nurses, and a multidisciplinary CPM team is more effective than management by a less diverse team [34]. Although the field of clinical pharmacology started late in China, as clinical pharmacist training has become widespread across the country, the participation of clinical pharmacists in the multidisciplinary management of cancer pain is becoming increasingly common. The value of pharmacist services urgently needs to be recognized by other clinical HCPs. A total of 54.65% of the respondents in this study indicated that there were clinical pharmacists participating in CPM, but only 19.69% of the hospitals or departments had a pharmacist to tumor patient ratio ≥1 : 50 (Supplementary Figure 2), indicating that there are too few clinical pharmacists in China. The levels of desire for input from pharmacists expressed by physicians, nurses, and pharmacists were significantly different (Table 1). Clinicians and nurses scored more than 70 points, while pharmacists scored only 64.76 ± 34.96 points, suggesting that physicians and nurses had stronger levels of desire for participation by clinical pharmacologists and valued their work more. Similarly, in terms of the demand for advanced management methods, nurses expressed a stronger desire for such methods than doctors and pharmacists (74.28 ± 36.05 vs. 70.84 ± 38.22, 57.23 ± 41.05, *P* < 0.01) (Table 1). In CPM, repeated pain assessments and the dose titration of analgesic drugs are often required, and pain assessments may take 0.5 to 20 minutes each time [35]. Due to the different roles in CPM played by physicians, pharmacists, and nurses, these professionals need to communicate frequently. All of these factors make the CPM complicated and time-consuming. In China, currently, there is no mobile management system for CPM that is widely used in clinical practice. Consequently, significant amounts of time would be saved and medical professionals' work efficiency would improve if an advanced management system could achieve the following functions: automatic screening of cancer pain patients, online pain assessments, intelligent delivery of pain orders, online conversions of opioid dosages, and online communication.

According to the stratified analysis of different occupational groups, professional title, hospital level, proportion of patient load accounted for by cancer pain patients, GPM ward evaluation status, number of annual trainings, and diversity of forms of training had different effects on the scores of the respondents. Presently, the literature on CPM practice, knowledge cognition, and attitude of physicians, pharmacists, and nurses is insufficient. Most of the established reports believe that the CPM knowledge of physicians or pharmacists is better than that of nurses, and nurses are better than physicians or pharmacists in pain assessment [16, 19, 25, 31]. In our present survey, the scores of pharmacists in CPM knowledge, practice, and attitude were all significantly lower than those of physicians and nurses, signifying that the performance of pharmacists was still worse than that of nurses. Only two studies dated over 3 decades ago agreed with the results of this survey [17, 18]. The reason for this may be that the positions of pharmacists involved in this investigation included clinical positions engaged in CPM and dispensing positions involved in cancer pain drug management. In China, the main population of hospital pharmacists are dispensing pharmacists. The pharmacists are responsible for the dispensing and management of narcotic drugs and generally do not participate in the clinical CPM work. Thus, they may lack the adequate knowledge of CPM. Only 29.20% of pharmacists involved in the survey came from medical oncology, surgical oncology, or pain departments, which led to the lower overall score of pharmacists. The result exemplifies the urgency and attentiveness needed to improve the level of CPM knowledge of pharmacists to better participate in the multidisciplinary management of cancer pain. Passing the GPM ward review and experiencing various training forms had significant positive impacts on the scores on all three parts of the survey in the three professions (*p*trend<0.05). Since 2011, China has started to engage in the GPM ward program nationwide to improve the management of cancer pain in China. This study confirmed for the first time that the creation of a GPM ward was very beneficial for increasing the practice, knowledge, and attitude of HCPs [24]. The effect of training on physicians, nurses, and pharmacists is not clear at present [16]. Several studies have reported that training can increase HCPs' knowledge, attitude, and practice regarding CPM [13, 35–37], while other studies found contrasting results [19, 38, 39]. In this study, training had a complex effect on the HCPs' scores (Figure 1). Here, 88.39% of the respondents received CPM training at least once a year, which was higher than the 30–65% reported in other countries [40,41], indicating that CPM training is relatively common in China. However, more training sessions do not necessarily mean a higher score, while more diverse forms of training were associated with have a higher probability of obtaining a high score (>65 points) (Figure 1). Hence, increasing only the number of trainings but using only one form of training may not be adequate to improve CPM. Diversifying the forms of training (e.g., case analysis, group discussions, concept discrimination, theory teaching, and practice guidance) may be an effective way of enhancing the quality of training. Surprisingly, there was no significant effect of professional title on the scores of HCPs, possibly due to the strengthening of CPM training and education in recent years [9], which has weakened the effect of work experience on CPM [18, 32, 42]. Consistent with the results of the study by Zhang et al. [14], the performance of HCPs from grade 3 first-level hospitals was better than that of HCPs from other grades of hospitals, which may also be partially due to the effect of training. Generally, grade 3 first-level hospitals have better HCP configurations, pay more attention to CPM, and provide HCPs with more opportunities to receive training. Moreover, the incidence of cancer pain in managed patients was negatively correlated with the knowledge scores of physicians and nurses. Contrary to the results of this study, other studies showed that the greater the proportion of HCPs' patient load accounted for by cancer pain patients, the higher the HCPs' level of CPM-related knowledge [19, 30, 43]. A possible reason for this is that, for a long time, Chinese physicians and nurses had a heavy workload [44, 45]. When managing many cancer pain patients, they did not have enough time to update their existing knowledge, even though their knowledge and attitude were incorrect.

When considering the results of this study, some important limitations must be taken into account. First of all, most of the CPM knowledge questions were subjective, and no comprehensive case questions (clinical scenarios) were used to test the true knowledge level of the HCPs. Second, although the options of hospital level and geographical location were set in the questionnaire, there was no requirement for participants to fill their working hospitals, so it was impossible to know how many hospitals were covered in this survey. Third, the scores of different types of questions had been corrected, which may have had an impact on the statistical analysis. Finally, the setting of the questions was not comprehensive, which hindered further in-depth assessment and analysis.

Although there were some defects in this survey, as a national cross-sectional survey, the sample size was up to 5012. These subjects came from all over the country and were evenly distributed in different levels of hospitals. Moreover, our findings were highly consistent with those of previous survey studies from China and abroad. Therefore, this survey could truly reflect the current situation of cancer pain management in China to a certain extent and provide reference for the improvement of cancer pain management.

## 5. Conclusions

In conclusion, HCPs' levels of practice and knowledge of CPM were average in China; however, pharmacists had the worst performance. The attitudes towards pharmacists' participation of HCPs showed that there is a need for pharmacists to participate in CPM, but faced with the short supply of pharmacists. Therefore, China urgently needs to strengthen the education and development of more competent clinical pharmacists to fully participate in the multidisciplinary management of cancer pain. In addition, we can try various training methods to enhance the quality and effect of HCP training, encourage hospitals to create GPM wards, and introduce advanced CPM methods to improve CPM success.

## Figures and Tables

**Table 1:** Scores of physicians, nurses, and pharmacists for each question and the *K*-*W* test of three groups.

Questionnaire questions	Scores (mean ± SD)		
	Physician	Nurse	Pharmacist
Practical ability of CPM^a,b,d,e^	57.00 ± 28.74	61.63 ± 28.99	50.04 ± 26.69
Q10. How often does your department follow-up with patients with cancer pain after discharge?^a,b,e^	46.18 ± 27.38	49.64 ± 28.54	44.33 ± 26.71
Q11. What are your most commonly used pain assessment methods or tools?^a,b,d,e^	46.34 ± 18.98	51.77 ± 19.14	43.62 ± 17.14
Q12. When will you conduct pain assessments for patients with cancer pain?^a,b,d,e^	55.80 ± 24.65	63.14 ± 26.94	45.35 ± 20.24
Q13. Before using analgesics, would you inform patients about the adverse reactions of the analgesics?^a,c,d,e^	79.67 ± 29.01	81.99 ± 28.26	66.85 ± 32.49
Cognition of CPM^a,b,d,e^	69.60 ± 28.45	64.28 ± 28.01	61.49 ± 28.95
Q16. What do you think of the analgesic treatment of patients with cancer?^a,d,e^	76.00 ± 24.40	75.46 ± 23.17	66.26 ± 24.52
Q17. Can you master the NCCN, ESMO or other authoritative guidelines, and use them to treat cancer pain patients?^a,b,d,e^	72.09 ± 28.28	65.03 ± 28.78	60.12 ± 29.78
Q18. Can you master the conversion of the equivalent dose of different opioids?^a,b,d^	73.69 ± 31.98	57.65 ± 34.74	59.17 ± 33.45
Q19. Do you know the principles of analgesic selection and dosage adjustment in patients with hepatorenal insufficiency?^a,b,d^	76.91 ± 27.57	67.53 ± 26.82	65.98 ± 29.03
Q20. Which of the following strong opioid adverse reactions are common?^a,b,d,e^	41.08 ± 18.61	44.59 ± 19.89	36.96 ± 17.77
Q21. Which of the following options are correct?^a,b,d,e^	77.85 ± 18.26	75.44 ± 18.16	80.43 ± 15.93
Attitudes towards pharmacists' participation and advanced methods in CPM^a,d,e^	70.35 ± 34.43	72.05 ± 33.71	62.07 ± 36.46
Q25. Will you consult a pharmacist if you encounter drug-related problems with regard to analgesic treatment?	68.37 ± 30.56	69.41 ± 31.94	68.08 ± 31.96
Q26. If not, why?^a,e^	24.44 ± 25.13	29.79 ± 24.60	18.46 ± 24.22
Q27. Do you think there is a need for pharmacists to participate in CPM?^a,d,e^	75.08 ± 32.08	76.05 ± 30.93	64.76 ± 34.96
Q29. Do you think it is necessary to introduce advanced CPM methods (e.g., a mobile CPM system) to improve the clinical management of cancer pain?^a,d,e^	70.84 ± 38.22	74.28 ± 36.05	57.23 ± 41.05

CPM, cancer pain management; NCCN, the National Comprehensive Cancer Network; ESMO, European Society for Medical Oncology; SD, standard deviation. ^a^*P* value of *K*-*W* test among the three groups <0.01; ^b^*P* value of *K*-*W* paired test (*α* = 0.05) between physician and nurse <0.01; ^c^*P* value of K–W paired test (*α*= 0.05) between physician and nurse <0.05; ^d^*P* value of *K*-*W* paired test (*α* = 0.05) between physician and pharmacist <0.01; ^e^*P* value of *K*-*W* paired test (*α* = 0.05) between nurse and pharmacist <0.01.

**Table 2:** Basic information of participants (*n* = 5012).

Characteristic	Physicians (*n* = 1279)	Nurses (*n* = 2267)	Pharmacists (*n* = 1466)	Overall (*n* = 5012)
Professional title				
Junior	538 (42.06)	1203 (53.07)	470 (32.06)	2211 (44.11)
Intermediate	558 (43.63)	856 (37.76)	812 (55.39)	2226 (44.41)
Senior	183 (14.31)	208 (9.18)	184 (12.55)	575 (11.47)

Area				
Central China	110 (8.60)	311 (13.72)	164 (11.19)	585 (11.67)
North China	542 (42.38)	746 (32.91)	802 (54.71)	2090 (41.7)
East China	226 (17.67)	261 (11.51)	162 (11.05)	649 (12.95)
South China	72 (5.63)	322 (14.20)	116 (7.91)	510 (10.18)
Northwest China	40 (3.13)	76 (3.35)	24 (1.64)	140 (2.79)
Northeast China	247 (19.31)	430 (18.97)	149 (10.16)	826 (16.48)
Southwest China	42 (3.28)	121 (5.34)	49 (3.34)	212 (4.23)

Hospital grade				
Below grade 2	25 (1.95)	130 (5.73)	70 (4.77)	225 (4.49)
Grade 2 second-level	35 (2.74)	73 (3.22)	77 (5.25)	185 (3.69)
Grade 2 first-level	169 (13.21)	327 (14.42)	341 (23.26)	837 (16.7)
Grade 3 second-level	290 (22.67)	396 (17.47)	408 (27.83)	1094 (21.83)
Grade 3 first-level	760 (59.42)	1341 (59.15)	570 (38.88)	2671 (53.29)

Departments				
Medical oncology	764 (59.73)	1370 (60.43)	119 (8.12)	2253 (44.95)
Surgical oncology	191 (14.93)	235 (10.37)	156 (10.64)	582 (11.61)
Pain	129 (10.09)	194 (8.56)	153 (10.44)	476 (9.5)
Anesthesiology	63 (4.93)	97 (4.28)	111 (7.57)	271 (5.41)
Other internal medicine	71 (5.55)	201 (8.87)	79 (5.39)	351 (7.00)
Other surgery	32 (2.50)	125 (5.51)	49 (3.34)	206 (4.11)
Pharmacy	29 (2.27)	45 (1.99)	799 (54.50)	873 (17.42)

Proportion of patients with cancer-related pain (for doctors and nurses only)				
0–20	325 (25.41)	510 (22.50)	N/A	835 (23.55)
21–40	455 (35.57)	732 (32.29)	N/A	1187 (33.47)
41–60	314 (24.55)	550 (24.26)	N/A	864 (24.37)
61–80	138 (10.79)	300 (13.23)	N/A	438 (12.35)
≥81	47 (3.67)	175 (7.72)	N/A	222 (6.26)

Passed GPM ward				
No	532 (41.59)	953 (42.04)	789 (53.82)	2274 (45.37)
Yes	747 (58.41)	1314 (57.96)	677 (46.18)	2738 (54.63)

Number of CPM trainings received per year				
0 time	165 (12.90)	210 (9.26)	207 (14.12)	582 (11.61)
1–3 times	667 (52.15)	1238 (54.61)	885 (60.37)	2790 (55.67)
4–6 times	296 (23.14)	509 (22.45)	276 (18.83)	1081 (21.57)
>6 times	151 (11.81)	310 (13.67)	98 (6.68)	559 (11.15)

Types of trainings on CPM				
0 type	23 (1.80)	55 (3.48)	51 (2.43)	129 (2.57)
1–2 types	766 (59.89)	1316 (77.22)	1132 (58.05)	3214 (64.13)
3–4 types	369 (28.85)	650 (15.21)	223 (28.67)	1242 (24.78)
≥5 types	121 (9.46)	246 (4.09)	60 (10.85)	427 (8.52)

Data are expressed as the *n* (%). GPM, good pain management; CPM, cancer pain management. N/A, not applicable.

**Table 3:** Proportions of physicians, nurses, and pharmacists with scores greater than 65 points and results of binary logistic regression analysis.

Characteristic	CPM practical ability		Knowledge of CPM		Attitudes towards pharmacists' participation and advanced methods in CPM	
	Proportion of people with scores >65 (%)	OR (95% CI)	Proportion of people with scores >65 (%)	OR (95% CI)	Proportion of people with scores >65 (%)	OR (95% CI)
Profession		^b^		^b^		^a^
Physician	33.15	1.00 (ref)	62	1.00 (ref)	65.21	1.00 (ref)
Nurse	44.38	1.77 (1.45–2.15)^b^	48.21	0.49 (0.41–0.57)^b^	64.93	0.86 (0.73–1.02)
Pharmacist	14.19	0.67 (0.42–1.07)	38.34	0.58 (0.40–0.83)^b^	47.75	0.6 (0.41–0.87)^a^
Professional title				^b^		^b^
Junior	37.4	1.00 (ref)	49.07	1.00 (ref)	66.4	1.00 (ref)
Intermediate	27.13	0.85 (0.71–1.01)	46	1.20 (1.04–1.38)^a^	53.59	0.81 (0.7–0.93)^b^
Senior	36	0.96 (0.74–1.25)	58.96	1.63 (1.31–2.04)^b^	60	0.79 (0.64–0.99)^a^
Area		_b_		_b_		_b_
Central China	47.86	1.00 (ref)	53.68	1.00 (ref)	73.5	1.00 (ref)
North China	12.3	0.47 (0.36–0.61)^b^	35.12	1.03 (0.83–1.29)	41.44	0.56 (0.44–0.7)^b^
East China	42.99	1.27 (0.95–1.69)	58.4	1.56 (1.21–2.02)^b^	71.65	1.19 (0.91–1.57)
South China	48.04	1.25 (0.92–1.69)	54.31	1.27 (0.97–1.67)	78.82	1.47 (1.08–1.99)^a^
Northwest China	62.14	2.07 (1.28–3.35)^b^	72.86	2.40 (1.52–3.77)^b^	83.57	1.76 (1.05–2.96)^a^
Northeast China	47.34	1.14 (0.87–1.51)	63.32	1.82 (1.42–2.33)^b^	69.25	0.97 (0.74–1.25)
Southwest China	47.17	1.18 (0.79–1.75)	56.6	1.35 (0.95–1.92)	72.64	1.01 (0.69–1.47)
Hospital grade		^a^		^b^		^b^
Below grade 2	15.11	1.00 (ref)	36.44	1.00 (ref)	48.89	1.00 (ref)
Grade 2 second-level	14.05	0.77 (0.41–1.45)	23.24	0.39 (0.24–0.62)^b^	38.38	0.77 (0.50–1.20)
Grade 2 first-level	22.7	1.13 (0.70–1.80)	37.99	0.69 (0.48–0.98)^a^	46.59	0.9 (0.64–1.27)
Grade 3 second-level	19.47	0.82 (0.51–1.31)	36.47	0.65 (0.46–0.92)^a^	45.8	0.96 (0.69–1.35)
Grade 3 first-level	43.99	1.18 (0.76–1.84)	60.13	1.05 (0.75–1.1.46)	72.41	1.52 (1.10–2.10)^a^
Department		^b^		^b^		^b^
Medical oncology	54.28	1.00 (ref)	63.78	1.00 (ref)	75.63	1.00 (ref)
Surgical oncology	13.57	0.35 (0.6–0.47)^b^	32.99	0.53 (0.42–0.66)^b^	40.89	0.58 (0.46–0.72)^b^
Pain	12.82	0.33 (0.24–0.46)^b^	32.56	0.62 (0.48–0.79)^b^	36.13	0.51 (0.40–0.64)^b^
Anesthesiology	8.12	0.21 (0.13–0.36)^b^	21.4	0.38 (0.27–0.53)^b^	26.94	0.37 (0.27–0.51)^b^
Other internal medicine	20.8	0.48 (0.35–0.67)^b^	38.18	0.74 (0.57–0.97)^a^	53.85	0.83 (0.64–1.09)
Other surgery	16.99	0.39 (0.25–0.61)^b^	30.58	0.52 (0.37–0.74)^b^	48.54	0.72 (0.52–1.01)
Pharmacy	16.61	0.44 (0.31–0.61)^b^	46.85	1.19 (0.93–1.15)	60.71	1.52 (1.19–1.95)^b^
Proportion of patients with cancer-related pain		^a^		^b^		^a^
0–20	34.13	1.00 (ref)	60.72	1.00 (ref)	70.54	1.00 (ref)
21–40	39.26	0.57 (0.38–0.85)^b^	55.6	1.73 (1.23–2.44)^b^	64.7	1.36 (0.95–1.94)
41–60	42.71	0.74 (0.50–1.09)	46.99	1.42 (1.02–1.98)^a^	60.19	1.13 (0.8–1.59)
61–80	45.89	0.82 (0.55–1.22)	47.26	0.93 (0.66–1.31)	64.84	0.94 (0.66–1.35)
≥81	49.1	0.76 (0.49–1.17)	47.75	0.86 (0.59–1.25)	65.32	1.05 (0.71–1.55)
Passed GPM ward		^b^		^b^		^b^
No	18.95	1.00 (ref)	34.17	1.00 (ref)	47.71	1.00 (ref)
Yes	44.08	2.16 (1.83–2.55)^b^	61.03	2.17 (1.90–2.48)^b^	70.16	1.81 (1.59–2.07)^b^
Number of CPM trainings received per year		^b^		^b^		^b^
0 time	9.11	1.00 (ref)	36.25	1.00 (ref)	50.52	1.00 (ref)
1–3 times	29.03	2.45 (1.76–3.43)^b^	48.42	1.39 (1.12–1.72)^b^	61.04	1.31 (1.06–1.61)^a^
4–6 times	38.76	3.24 (2.25–4.67)^b^	47.09	1.17 (0.91–1.50)	56.15	1.02 (0.80–1.3)
>6 times	63.69	5.23 (3.48–7.84)^b^	67.44	1.79 (1.34–2.40)^b^	71.91	1.15 (0.86–1.55)
Types of trainings on CPM		^b^		^b^		^b^
0 type	10.85	1.00 (ref)	24.81	1.00 (ref)	46.51	1.00 (ref)
1–2 types	15	1.04 (0.56–1.93)	37.37	1.67 (1.07–2.62)^a^	48.72	1.27 (0.86–1.89)
3–4 types	63.12	5.74 (3.07–10.73)^b^	69.24	4.48 (2.81–7.14)^b^	79.23	3.11 (2.05–4.74)^b^
≥5 types	83.84	13.77 (7.02–27.00)^b^	83.14	8.24 (4.89–13.88)^b^	92.74	8.89 (5.15–15.36)^b^

GPM, good pain management; CPM, cancer pain management; OR, odds ratio; CI, confidence interval; ref, reference. ^a^*P* < 0.05; ^b^*P* < 0.01.

## Data Availability

The datasets used and/or analysed during the current study are available from the corresponding author upon reasonable request.

## Supplementary Materials

Supplementary Materials Supplementary Figure 1. The analgesic drugs most commonly used by physicians and the reasons for their use (*n* = 1279). Supplementary Figure 2. Pharmacists' participation in CPM (*n* = 5012) and the ratio of pharmacists involved in CPM to cancer patients (*n* = 2739). Supplementary Figure 3. The responsibilities of pharmacists in CPM (*n* = 2739). Supplementary Figure 4. The functions of CPM methods expected by participants (*n* = 5012). The result of each option was the sum of the proportions of doctors, nurses, and pharmacists in their respective occupational groups.
