# Supplementary material for: Practice, Knowledge, and Attitude of Health Care Providers regarding Cancer Pain Management: A National Survey
Source: Pain Res Manag. 2021 Aug 23;2021:1247202. doi: 10.1155/2021/1247202 (PMC8405340; doi:10.1155/2021/1247202)
Supplement: Supplementary Materials — Supplementary Figure 1. The analgesic drugs most commonly used by physicians and the reasons for their use (n = 1279). Supplementary Figure 2. Pharmacists' participation in CPM (n = 5012) and the ratio of pharmacists involved in CPM to cancer patients (n = 2739). Supplementary Figure 3. The responsibilities of pharmacists in CPM (n = 2739). Supplementary Figure 4. The functions of CPM methods expected by participants (n = 5012). The result of each option was the sum of the proportions of doctors, nurses, and pharmacists in their respective occupational groups. [file 1247202.f1.docx]

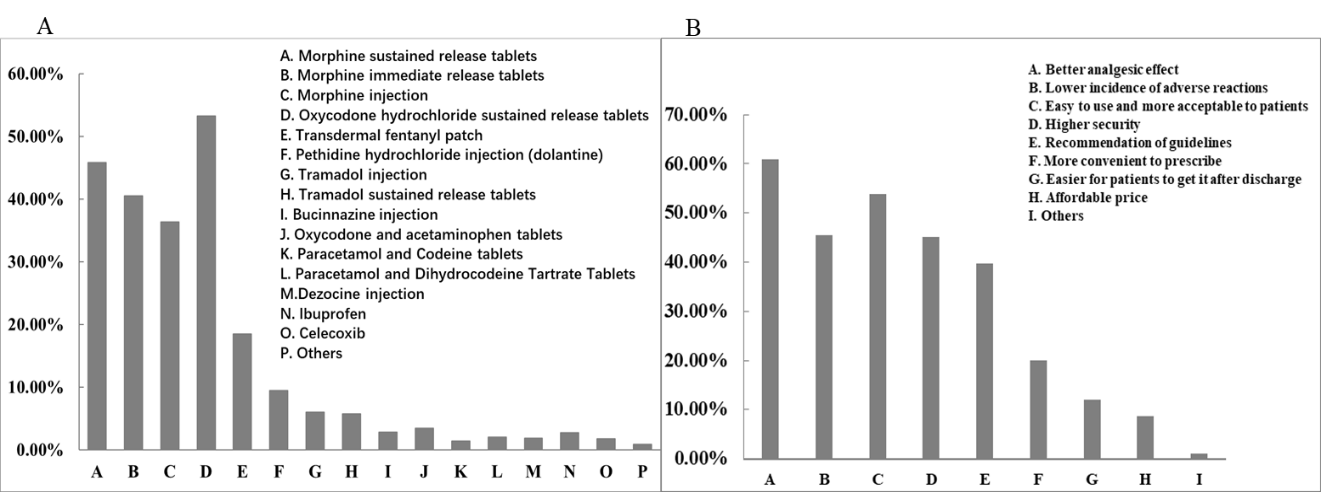
Figure 1: The analgesic drugs most commonly used by physicians and the reasons for their use (n=1279).


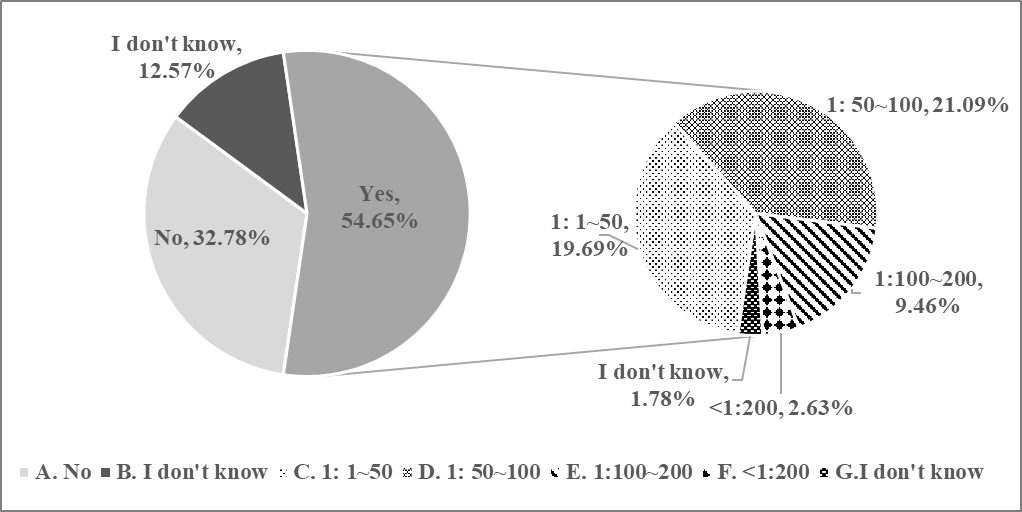


Figure 2: Pharmacists' participation in CPM (n=5012), and the ratio of pharmacists involved in CPM to cancer patients (n=2739).

**
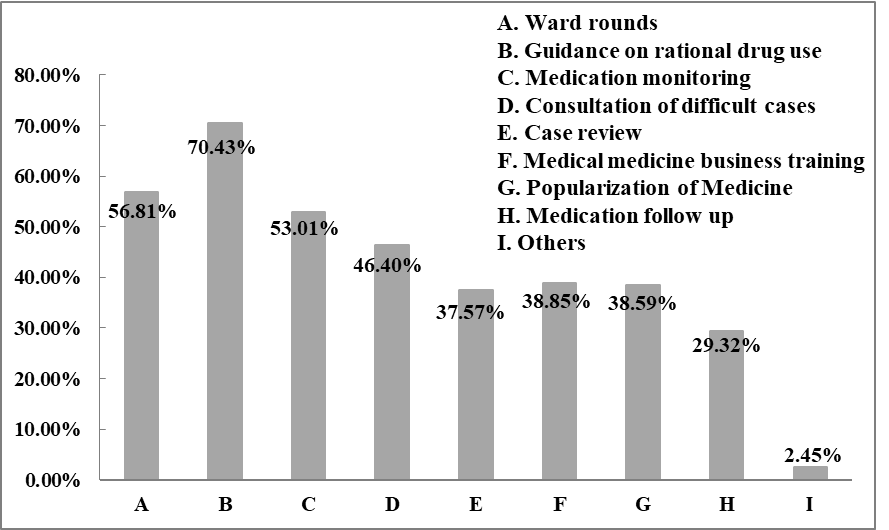
**

Figure 3: The responsibilities of pharmacists in CPM (n=2739).

**
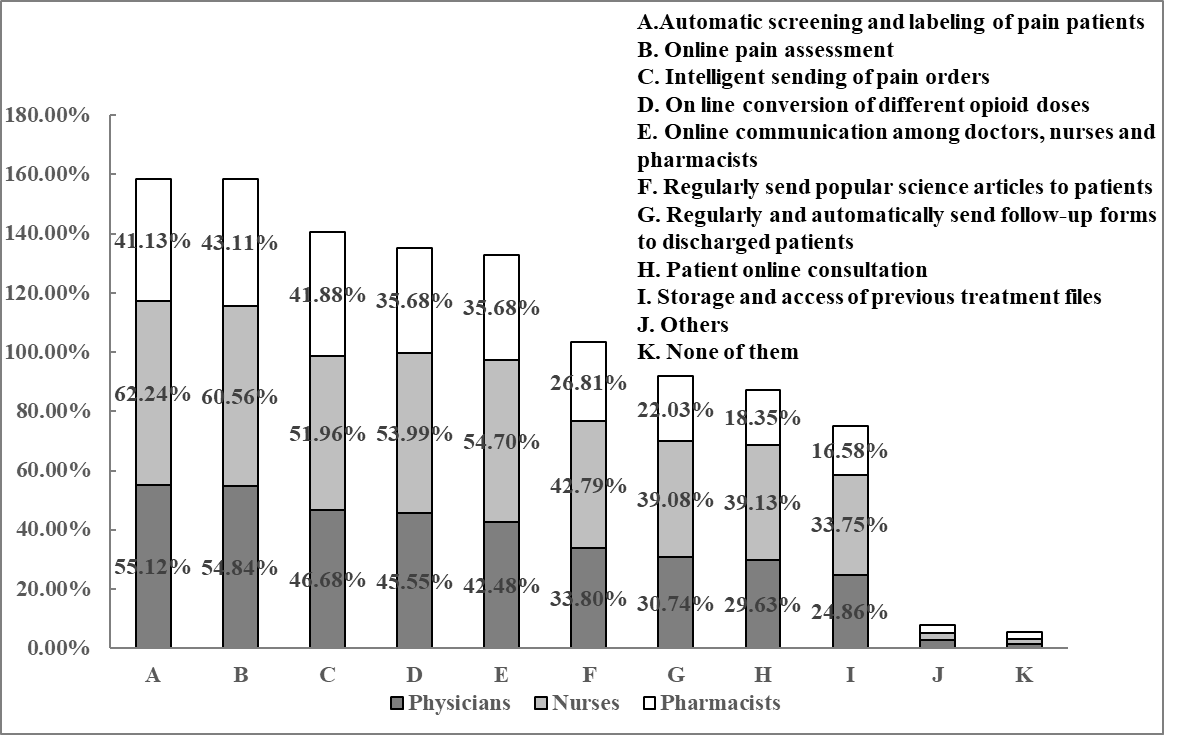
**

Figure 4: The functions of CPM methods expected by participants (n=5012). The result of each option was the sum of the proportions of doctors, nurses and pharmacists in their respective occupational groups.
